# Dermoscopic Features of Cutaneous Endometriosis Arising in a Cesarean Scar: A Case Report

**DOI:** 10.1155/crdm/6880602

**Published:** 2024-12-31

**Authors:** Kevin Yang, Karim Saleh

**Affiliations:** Division of Dermatology and Venereology, Department of Clinical Sciences, Skåne University Hospital, Lund University, Lund, Sweden

**Keywords:** caesarean section, cutaneous endometriosis, dermoscopy, scar

## Abstract

Cutaneous endometriosis is a rare manifestation of endometriosis, and few reports on its dermoscopic features have been published. In this case report, we present a 40-year-old female with cutaneous endometriosis arising in a caesarean scar, exhibiting unique and distinct dermoscopic features. The patient presented with a nodular, papillomatous growth in the right end of the scar, and dermoscopic examination revealed structureless red papillomatous projections, as well as nonpapillomatous areas with red dotted vessels surrounded by a white reticular network. A biopsy confirmed the diagnosis of endometriosis. To our knowledge, this is the first report of such dermoscopic features in cutaneous endometriosis arising in a caesarean scar. Our case report adds to the current limited knowledge of dermoscopic features of cutaneous endometriosis and may help in the diagnosis of this condition.

## 1. Introduction

Cutaneous endometriosis is a rare medical condition characterized by the growth of an endometrial tissue on the skin outside of the uterus. Dermoscopic examination of cutaneous endometriosis can provide valuable information for its diagnosis. However, only a few reports on its dermoscopic features have been published, and most of these mainly pertain to cutaneous endometriosis in the umbilicus. Here, we report a case of cutaneous endometriosis arising in a caesarean scar, with unique and distinct dermoscopic features.

## 2. Case Presentation

**Figure 1:**
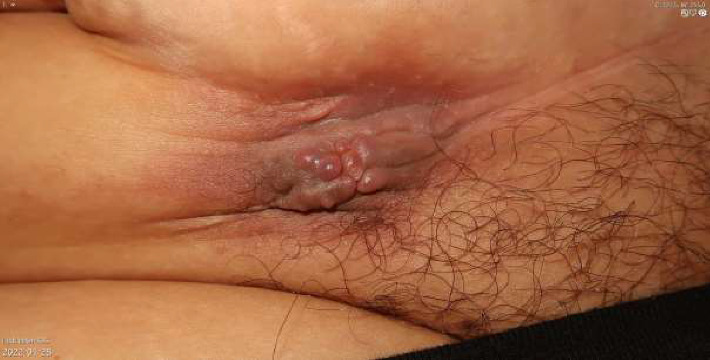
Clinical photograph of tumorous growth arising in the cesearean scar.

**Figure 2:**
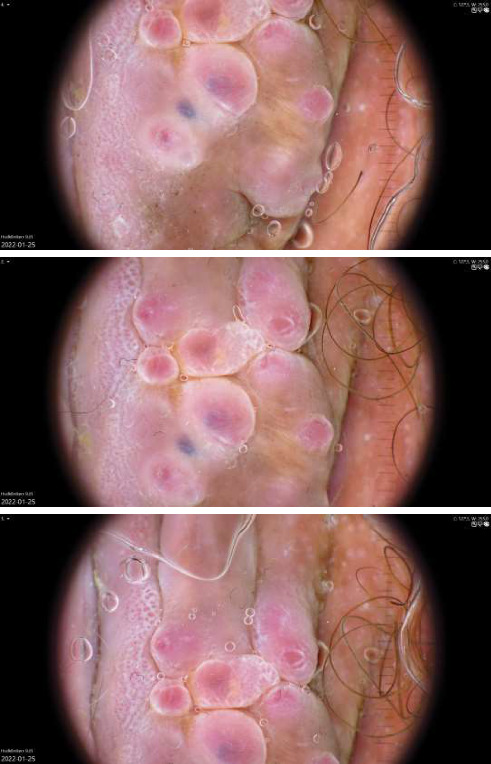
Polarized contact dermoscopy of the lesion.

**Figure 3:**
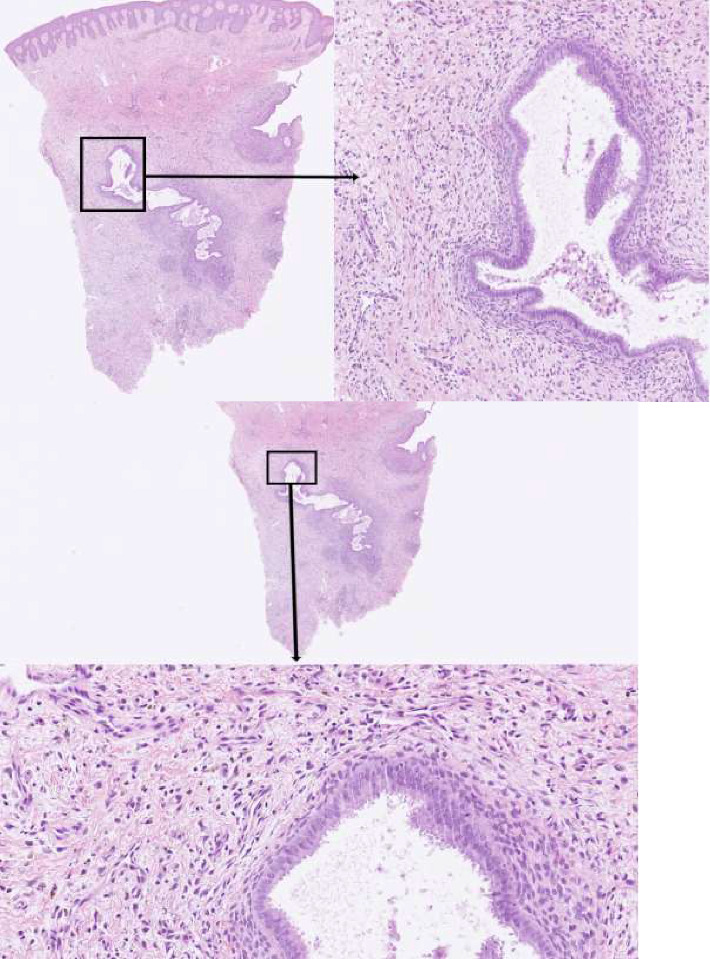
Histopathological examination demonstrates the combination of endometrial glands and stroma in a biopsy specimen obtained from the lesion. The endometrial glands show a secretory phase (with decapitation secretion).

A 40-year-old female with Fitzpatrick skin type 3 presented to our clinic with a tumorous growth in a caesarean scar. The patient was healthy and had no history of previous dermatological conditions. The patient had undergone two caesarean sections with the most recent one 5 years ago and had experienced no postoperative complications. Two years after the last caesarean section, the patient noticed a small, painful tumorous growth on the right end of the scar that slowly grew to 4 cm in size. Macroscopically, the lesion was nodular with papillomatous growths on top (Figure 1). Dermoscopic examination (Figure 2) revealed structureless red papillomatous projections, as well as nonpapillomatous areas with red dotted vessels encompassed by a white halo in a reticular network-configuration. At higher magnifications, structureless red on the papillomatous projections was discerned to be composed of dotted vessels. In addition, few purple clods could be seen. Upon further questioning, the patient confirmed cyclical bleeding from the tumor that corresponded with her menstrual cycle. A punch biopsy confirmed the diagnosis of endometriosis in the luteal phase (Figure 3). The patient was referred to a gynecological clinic and is now scheduled for surgery.

## 3. Discussion

To our knowledge, only a few reports on the dermoscopic features of cutaneous endometriosis have been published (Table 1), with most of these reports mainly pertaining to cutaneous endometriosis in the umbilicus. Only one article could be found discussing cutaneous endometriosis in caesarean scars, as in our report. In contrast to our dermoscopic findings, the only other cutaneous endometriosis in a caesarean scar described by Tognetti et al. had dermoscopic findings of pigmented arborizing lines and granular structures over a gray background [5]. It also had bright dots and targetoid brownish structures. None of these features could be seen in our patient's lesion.

Also differing from our findings, Jaimie et al. described a lesion consisting of an area of homogeneous structureless reddish localized pigmentation, a brown amorphous area, and parts where the normal skin network was more evident [2]. These lesions were different from our patient's lesion both macroscopically and dermoscopically.

Resembling the lesion of our patient, Costa et al. described a lesion with erythematous violaceous polypoid projections with light brown round spots and areas with bleeding in the follicular phase with an increased volume of erythematous-bluish in the luteal phase [3]. The violaceous parts described by Costa et al. are much more distinct in our case; however, upon closer inspection, they are better described as purple clods. Secondly, these purple clods are not limited to only polypoid projections in our case. Due to their color, we believe these clods to be of pools of deoxygenated blood. Additionally, no dotted vessels could be discerned in the case presented by Costa et al. Conversely, these dotted vessels were reported by De Giorgi et al. as homogeneous red pigmentation with embedded red globules, gradually fading toward the periphery [1], while Buljan et al. reported homogeneous dotted vessels over a red structureless area with brownish hue [4]. However, neither of these reports presented a distinctive case of dotted vessels encompassed by a white negative reticular network, as we do. Furthermore, Di Georgi et al.'s and Buljan et al.'s dermoscopic findings were of umbilical lesions as compared to our caesarian section.

The primary strength of this case report is that it provides dermoscopic pictures with a detailed description of a condition scarcely described in the literature. This may also raise awareness of this rare condition. It is also distinctively different from the few previously published case reports. The main limitation of this case report is that it consists of a single case, which may limit the generalizability, as it is currently unknown what other appearances of cutaneous endometriosis can be assumed. Additional studies are needed to confirm our findings.

## 4. Conclusion

Our case report adds to the current limited knowledge of the dermatological features of cutaneous endometriosis. The distinctive dermoscopic features we described, primarily dotted vessels encompassed by a white reticular network, have not been previously reported. This report also raises awareness of the rarity of this condition. Additional case reports and studies are needed to better understand the appearance and characteristics of cutaneous endometriosis at various locations on the skin.

## Figures and Tables

**Table 1:** Dermoscopic features of cutaneous endometriosis in previously published case reports, as well as the dermoscopic features of umbilical cutaneous endometriosis in our case report.

Case report	Dermoscopic features
De Giorgi et al. [1]	Brownish, elevated, dome-shaped, bilobular nodule. Dermoscopy showed homogeneous reddish pigmentation, regularly distributed, gradually fading toward the periphery. Within this typical pigmentation, there were small red globular structures but more defined and of a deeper hue, which the authors called ‘red atolls'
	⁣^∗^Multilobulated red nodular lesion with homogenous distributed red clods between white branching/reticular network

Jaime et al. [2]	Shiny reddish-brown nodule. Dermoscopy showed homogeneous reddish localized pigmentation, with no differentiated structures. In addition, an amorphous brown area was present, where a normal skin network was more evident in some parts
	⁣^∗^Multilobulated nodular lesion. One lobule with structureless brown pigmentation. One lobule with a reticular brown network and another lobule with structureless pink color

Costa et al. [3]	Nodular lesion. Follicular phase: Polypoid projections of erythematous violaceous color, area with dark brown spots and area of active bleeding. Luteal phase: Increased volume of polypoid projections with erythematous to bluish color, increase in the number and size of dark brown spots, and areas with dark material
	⁣^∗^Pink nodular lesion with polypoid projections. Follicular phase: Violaceous polypoid projections of erythematous next to areas with dark brown dots. Areas with active bleeding. Luteal phase: Additional number of polypoid projections compared to the follicular phase. Darker brown pigmentation in areas with dots conflating into irregular structures' brown areas

Buljan et al. [4]	Nodular lesion with diffused and homogeneously distributed dotted vessels over a milky red structureless area with a brownish hue
	⁣^∗^Red nodular lesion with homogeneously distributed dotted vessels over a light red structureless background

Tognetti et al. [5]	Brown nodule with a papillomatous surface. Dermoscopy showed alternation of intensely pigmented arborizing and granular structures over a gray background. At higher magnification “branches and fruits” appearance could be seen with pigmented arborizing lines combined with pigmented dots. The lines assumed a parallel disposition within the scar portion. Bright shiny points and targetoid brownish structures could be seen
	⁣^∗^Brown branched lines and dots over a grayish background. The lines assume a parallel configuration within the scar portion. Few shiny dots

Our case	Structureless red papillomatous projections and nonpapillomatous areas with red dotted vessels with encompassing white halo in a white reticular network configuration. Additionally, a few purple clods could be identified. At higher magnifications, the structureless red areas on the papillomatous projections were discerned to be composed of dotted vessels as well

*Note:* The dermoscopic descriptions used in each article are provided. For more homogenous use of dermoscopic terminology, we also provide corresponding dermoscopic descriptions based on our analysis of the published dermoscopic pictures from each publication (⁣^∗^). For original pictures, please refer to each individual case report.

## Data Availability

The data that support the findings of this study are available from the corresponding author upon reasonable request.
